# Chronic exposure to sublethal concentrations of Boral® 500 SC (sulfentrazone) induces sex- and age-dependent metabolic and behavioral effects in *Drosophila melanogaster*

**DOI:** 10.1007/s11356-026-37953-z

**Published:** 2026-06-23

**Authors:** Mateus Cristofari Gayer, Maria Elizabeth Gomes Paz, Murilo Ricardo Sigal Carriço, Nicolas Gabriel Becker Flores, Elton Luis Gasparotto Denardin, Clésio Soldateli Paim, Rafael Roehrs, Robson Luiz Puntel

**Affiliations:** 1 Post-Graduation Program in Biochemistry, Federal University of Pampa (UNIPAMPA) - Campus Uruguaiana, BR-472 Km 7, CEP 97508-000 Uruguaiana, Rio Grande Do Sul Brazil; 2 Post-Graduation Program in Pharmaceutical Sciences, Federal University of Pampa (UNIPAMPA) - Campus Uruguaiana, BR-472 Km 7, CEP 97508-000 Uruguaiana, Rio Grande Do Sul Brazil

**Keywords:** Ecological risk assessment, Herbicide toxicity, Non-target organism, Aging, Sexual dimorphism

## Abstract

Sulfentrazone (SULF) is a widely used herbicide characterized by environmental persistence and potential off-target dispersion, yet its chronic sublethal effects on insects remain insufficiently understood. Because conventional toxicity assessments often prioritize lethality endpoints, they may overlook functional impairments that compromise organismal fitness. Here, we used *Drosophila melanogaster* as an integrative insect model to evaluate whether chronic exposure to sublethal concentrations of the commercial formulation Boral® 500 SC (SULF) affects development, female reproduction, locomotor performance, metabolic status, longevity, and stress resistance. The herbicide did not significantly affect developmental parameters, but significantly reduced longevity in both female and male flies. Moreover, exposure impaired climbing locomotor performance and exacerbated age-related functional decline. In females, a significant reduction in body weight was observed at 7 days of age, along with decreased carbohydrate levels at 30 days, whereas males exhibited increased lipid accumulation at 30 days of age. Oviposition rate was significantly reduced at 0.25 mg/L and 0.5 mg/L, and females exposed to 0.5 mg/L also displayed reduced resistance to paraquat-induced oxidative stress. Principal component analysis (PCA) revealed significant correlations between metabolic and behavioral parameters, highlighting the central role of energy reserves in mediating physiological and behavioral dysfunctions. Collectively, these findings demonstrate that Boral® 500 SC induces biologically relevant sublethal effects in *D. melanogaster*, suggesting that current regulatory methodologies may substantially underestimate the ecological risks of SULF to insects, thereby potentially contributing to population declines in non-target species.

**Graphical abstract:**

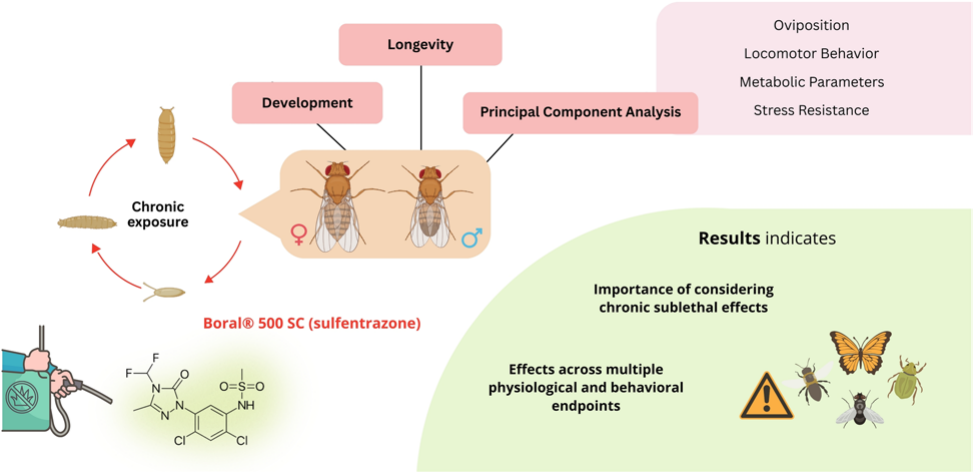

## Introduction

The widespread use of herbicides has raised growing concern regarding their environmental impacts on both terrestrial and aquatic ecosystems. Moreover, toxicological risk assessments conducted by regulatory agencies may not always be sufficiently comprehensive to support effective policies aimed at mitigating the damage caused by the application of agrochemicals in modern agricultural practices (Möhring et al. 2020). Current regulatory frameworks frequently rely on a limited set of endpoints, with a strong emphasis on acute mortality, while sublethal and chronic effects that directly affect the ecological fitness of non-target organisms are often overlooked (Straub et al. 2020). In addition, standard toxicity testing protocols commonly focus on isolated active ingredients, disregarding the potential interactive effects between active compounds and formulation adjuvants present in commercial products (Mesnage and Antoniou 2018; Straw and Brown 2021). Together, these limitations underscore the need for ecotoxicological studies that incorporate integrative biological endpoints, allowing for a more realistic, life-stage-sensitive, and ecologically relevant assessment of herbicide impacts on non-target species.

In this context, the herbicide SULF has been classified as “practically non-toxic” to the honey bee *Apis mellifera* by the U.S. Environmental Protection Agency (US EPA), based exclusively on acute oral and contact toxicity assays performed in adult individuals (Aufderheide and Kranzfelder 1996; Sinclair et al. 2014). Such evaluations typically assess short-term mortality following exposure to the isolated active ingredient, usually in a single insect species, thereby providing a narrow representation of potential ecological risks, with limited relevance for predicting chronic or sublethal outcomes across insect taxa. To date, the scientific literature remains remarkably scarce regarding the toxicological effects of SULF on insects, apart from our previous study using *D. melanogaster*, in which acute exposure to high concentrations of the commercial formulation Boral® 500 SC induced classical symptoms consistent with its known mode of action, accompanied by reduced survival and impaired locomotor performance (Gayer et al. 2025). Furthermore, accumulating evidence indicates that SULF and its commercial formulations can exert biologically relevant effects on a range of non-target organisms, including tadpoles (Freitas et al. 2017; Wilkens et al. 2019; da Silva et al. 2021), earthworms (Mesak et al. 2018; Li et al. 2020), and zebrafish (Jiang et al. 2022; Wang et al. 2024), even at low exposure levels. Importantly, environmental monitoring studies have reported the presence of SULF in soils and surface runoff waters at sites where the herbicide is applied in accordance with regulatory guidelines, with concentrations reaching up to 318 ng/g dry weight in soil and 10.3 µg/L in runoff water (Thorngren et al. 2017).

SULF is a protoporphyrinogen oxidase (PPOX) inhibitor, an enzyme involved in the biosynthesis of the porphyrin ring, a structural component of chlorophylls and heme, which are highly conserved molecules across plants and animals (Zagar et al. 2019). Given this evolutionary conservation, inhibition of PPOX raises concerns regarding unintended effects on non-target animal species, reinforcing the importance of expanding knowledge on the ecological and physiological consequences of SULF exposure in organisms inhabiting agricultural landscapes. This concern is particularly relevant for insects, which play critical roles in ecosystem functioning, constitute a major component of global biodiversity, and are increasingly threatened by anthropogenic stressors, including pesticide exposure (Sánchez-Bayo and Wyckhuys 2019; Raven and Wagner 2021). Within this framework, *Drosophila melanogaster* emerges as a highly informative model organism for ecotoxicological research, due to its ease of laboratory maintenance, short life cycle, and extensive genetic, physiological, and behavioral characterization accumulated over more than a century of scientific use (Schneider 2000; Wāng 2025). By enabling the integration of developmental, behavioral, metabolic, reproductive, and stress-related endpoints across the lifespan, this model provides a sensitive and mechanistically informative platform for detecting sublethal toxic effects with potential ecological relevance.

By addressing chronic sublethal exposure to a commercial SULF formulation in an insect model, this work aims to expand current understanding of the functional consequences of herbicide exposure beyond animal mortality endpoints. Therefore, this study was designed to test the hypothesis that chronic exposure to sublethal concentrations of the commercial formulation Boral® 500 SC (0.1, 0.25, and 0.5 mg/L) induces biologically relevant sublethal effects, manifested as disruptions in development, female reproductive output, locomotor performance, metabolic homeostasis, and resistance to stress in *D. melanogaster*. In this context, these findings may provide evidence to support more comprehensive ecological risk assessment strategies and to refine approaches aimed at mitigating herbicide-related impacts on non-target organisms.

## Materials and methods

### Chemicals

The commercial formulation of the SULF herbicide used in this study was Boral® 500 SC (FMC Corporation), containing 50% (m/v) SULF and 70% (m/v) formulation additives. This product is registered with the Brazilian Ministry of Agriculture, Livestock, and Food Supply (MAPA) under registration number 07495.

Chemical reagents, including bovine serum albumin (BSA; CAS No.: 9048–46-8), Coomassie Brilliant Blue G (CAS No.: 6104–58-1), methylparaben (CAS No.: 99–76-3), methyl viologen dichloride hydrate (paraquat; CAS No.: 75365–73-0), and vanillin (CAS No.: 121–33-5), were purchased from Sigma-Aldrich (St. Louis, MO, USA). Agar (CAS No.: 9002–18-0), anhydrous glucose (CAS No.: 50–99-7), anthrone (CAS No.: 90–44-8), and sucrose (CAS No.: 57–50-1) were purchased from Dinâmica Química (Indaiatuba, SP, Brazil). Orthophosphoric acid (CAS No.: 7664–38-2) and sulfuric acid (CAS No.: 7664–93-9) were purchased from CRQ Química (Diadema, SP, Brazil). Other materials used for the maintenance of the *D. melanogaster* stock were obtained from local commercial suppliers.

### *Drosophila melanogaster* stock

Wild-type *D. melanogaster* (Harwich strain) was obtained from the National Species Stock Center (Bowling Green, USA). Flies were reared and maintained on a standard cornmeal-based diet composed of 11.12% (m/v) coarse cornmeal, 8.9% (m/v) medium cornmeal, 2.22% (m/v) sucrose, 2.22% (m/v) wheat germ, 0.32% (m/v) powdered milk, 0.25% (m/v) brewer’s yeast, 0.15% (m/v) sodium chloride, and 0.08% (m/v) methylparaben. Cultures were maintained under controlled temperature of 24 ± 1 °C, ambient relative humidity and light/dark cycle, until the onset of experimental treatments.

### Experimental design: exposure to Boral® 500 SC

Adult *D. melanogaster* pairs (≤ 4 days old) were introduced into disposable Petri dishes containing a standard agar-based medium (1% agar, 1% sucrose, 1% milk powder, and 0.08% methylparaben, w/v) and allowed to oviposit for 24 h. After this period, adults were removed, and the eggs were collected and transferred to culture vials containing the same standard diet, either unsupplemented (control) or supplemented with the commercial formulation Boral® 500 SC at nominal concentrations of 0.10, 0.25, or 0.50 mg/L a.i.

The concentrations used in the present study were selected based on the limited environmental occurrence and toxicological reference data available for SULF. Field monitoring studies have reported SULF concentrations in agricultural soils of up to 318 ng/g dry weight following sequential applications under realistic agricultural conditions, although direct comparison with our exposure levels is constrained by differences in matrices and reporting units (Thorngren et al. 2017). Additionally, SULF exhibits a relatively long soil half-life (146.5 days) (Martinez et al. 2008), supporting the environmental relevance of prolonged exposure scenarios. The U.S. EPA Aquatic Life Benchmarks establish a chronic toxicity reference concentration of 0.2 mg/L for aquatic invertebrates, providing one of the few chronic toxicity benchmarks available for non-target invertebrate species. Furthermore, the selected concentrations are supported by previous toxicity studies demonstrating that this exposure range remains below acute lethal thresholds in *D. melanogaster* (Gayer et al. 2025). Therefore, the concentrations employed in the present study were chosen to represent low, sublethal exposure levels under a chronic exposure scenario, taking into account the available environmental occurrence data, the persistence of SULF in soil, existing chronic toxicity benchmarks, and prior toxicological evidence.

The flies were kept in a Panasonic MIR-204-PA laboratory incubator (Japan) under controlled conditions, constant temperature of 24 ± 0.5 °C, 12 h light/dark photoperiod, and ambient relative humidity. Illumination was provided by a 15 W cool-white fluorescent lamp (6400 K), with light intensity maintained at 300 ± 20 lx, as measured using a Minipa MLM-1011 digital lux meter (Brazil).

### Development, longevity and oviposition

To assess the effects of herbicide on development and longevity, transferred eggs were monitored daily. Based on these observations, the following endpoints were determined: (i) larval survival rate; (ii) pupation rate, defined as the proportion of larvae reaching the pupal stage; (iii) adult emergence rate, defined as the proportion of pupae successfully emerging as adults; and (iv) total number of adults per vial. Fifteen eggs were allocated per vial, with ten vials per experimental group (*n* = 10, totaling 150 eggs per group). In addition, newly emerged adults were transferred to fresh vials containing the corresponding herbicide concentrations or control medium for longevity assessment. The standard agar-based medium was renewed three times per week, during which mortality and oviposition were recorded. This monitoring continued until the death of all individuals.

### Locomotor behavior

Locomotor behavior was assessed in adult *D. melanogaster* that developed from the embryonic stage under continuous exposure to either the commercial formulation Boral® 500 SC or control medium. Behavioral assays were conducted in flies according to sex (males and females) and age (7 or 30 days post-emergence) for each herbicide concentration and the control group.

#### Negative geotaxis assay

The assessment of climbing ability was performed using the negative geotaxis assay (Sudati et al. 2013), with minor modifications. Briefly, groups of 3–5 adult flies were transferred to vertical glass columns (20 cm in height × 2 cm in diameter). Following a 15-min recovery period after ice anesthesia, insects were gently tapped to the bottom of the column, and the time required for each individual to reach a height of 15 cm was recorded. For each column, three consecutive trials were conducted, and the mean climbing performance for each individual was calculated. The sample size for each experimental subgroup (sex/age/treatment) ranged from 15 to 22 individuals (*n* = 15–22).

#### Open field locomotion assay

Exploratory locomotor activity was assessed using an open-field arena assay, as originally described by Feany and Bender (2000). Flies were briefly anesthetized on ice and subsequently placed in a circular arena (9 cm diameter) subdivided into 1 × 1 cm squares, covered with a Petri dish lid, with three flies per arena. Following a 30-min acclimation period to allow recovery from anesthesia, locomotor activity was recorded using a cell phone camera. The number of grid squares crossed (locomotor crossings) by each individual was quantified during a 30-s observation period. The sample size for each experimental subgroup (sex/age/treatment) ranged from 25 to 35 individuals (*n* = 25–35).

### Metabolic parameters

Following completion of the behavioral assays, stored flies were allocated to pre-weighed microcentrifuge tubes, with 3–5 individuals per tube. Tubes were reweighed, and the difference between measurements was used to determine total body mass. Subsequently, flies were homogenized with an IKA T10 Basic homogenizer (Germany) in distilled H_2_O, and total protein, carbohydrate, and lipid contents were quantified. For each experimental group, including the different herbicide concentrations and the control, nine to eleven microtubes were prepared (*n* = 9–11, 30 to 45 total individuals per group), with samples segregated by sex and age. For all tests, three technical replicates were analyzed for each sample, absorbance was measured using a Biochrom EZ Read 2000 microplate reader (United Kingdom), and results are expressed as the mean of these replicates. Biochemical parameters were normalized to the total body mass of each sample to account for differences in body size.

#### Total protein levels

Total protein content was quantified using the Bradford assay (Bradford 1976). Briefly, 5 µL of each sample homogenate was dispensed into microplate wells, followed by the addition of 45 µL of distilled H_2_O and 200 µL of Coomassie Brilliant Blue G reagent, freshly prepared according to the manufacturer’s instructions. Bovine serum albumin (BSA) was used as the protein standard for construction of the analytical calibration curve under absorbance measured at 595 nm.

#### Total carbohydrate levels

Total carbohydrate content was quantified using the anthrone colorimetric method (van Handel 1985a). Briefly, 25 µL of each sample homogenate was transferred to glass tubes, followed by the addition of 1,000 µL of anthrone reagent (0.14% w/v anthrone in 70% v/v H₂SO₄). The tubes were incubated in a water bath at 90 °C for 17 min, then allowed to cool to room temperature. Subsequently, 250 µL aliquots were transferred to microplate wells, and absorbance was measured at 625 nm. Glucose was used as the standard for construction of the analytical calibration curve.

#### Total lipid levels

Total lipid content was quantified using the vanillin–phosphoric acid colorimetric method (van Handel 1985b). Briefly, 25 µL of each sample homogenate was transferred to glass tubes containing 175 µL of concentrated H_2_SO_4_. The tubes were incubated in a water bath at 90 °C for 20 min and subsequently cooled to room temperature. Then, 500 µL of vanillin–phosphoric acid reagent (0.14 g/100 mL) was added, and the tubes were incubated in the dark to allow color development at room temperature. Following this step, 250 µL aliquots were transferred to microplate wells, and absorbance was measured at 525 nm. Soybean oil was used as the standard for construction of the analytical calibration curve.

### Analysis of stress resistance

To evaluate resistance to multiple environmental stressors, flies were stratified by sex (females and males) and age (7 and 30 days post-emergence). Three independent assays were performed: (i) desiccation resistance, in which flies were transferred to empty vials without access to food or water; (ii) starvation resistance, where flies were provided with 5 mL of 1% agar as a moisture source in the absence of nutrients; and (iii) oxidative stress resistance, in which animals were maintained on 5 mL of standard agar-based diet supplemented with 20 mM paraquat (Lin et al. 2023). For each assay, 30–40 total individuals per subgroup (age/sex/treatment) were used and distributed across at least five 50 mL Falcon tubes to minimize potential overcrowding effects (*n* = 30-40). Mortality was recorded at 2-h intervals, and results are expressed as the percentage of surviving flies over time.

### Statistical analysis

Statistical analyses and graphical representations were performed using GraphPad Prism software (version 8.0.1). Data distribution normality was assessed prior to analysis. When appropriate, datasets were analyzed using one-way or three-way analysis of variance (ANOVA), considering age, sex, and herbicide concentration as fixed factors, followed by Tukey’s multiple-comparison post hoc test. Survival (longevity) data were analyzed using the log-rank (Mantel–Cox) test for trend. For stress resistance assays, the area under the survival curve (AUC) was calculated and subsequently analyzed using three-way ANOVA. Data are presented as mean ± standard error of the mean (SEM) or as individual data points, as appropriate. Statistical significance was defined as *P* < 0.05, and the specific statistical tests applied to each dataset are indicated in the respective figure legends.

Principal components analysis (PCA) was conducted on normalized and standardized datasets encompassing reproductive, behavioral, metabolic, and stress-related endpoints in *D. melanogaster* chronically exposed to Boral® 500 SC. Analyses were performed using Origin 8 (v.8.0). Biplots of the first two principal components (PC1 and PC2) were generated to illustrate correlations among variables across sex and age groups.

## Results

### Effects of Boral® 500 SC (SULF) on development, adult longevity, and oviposition in *D. melanogaster*

**Fig. 1:**
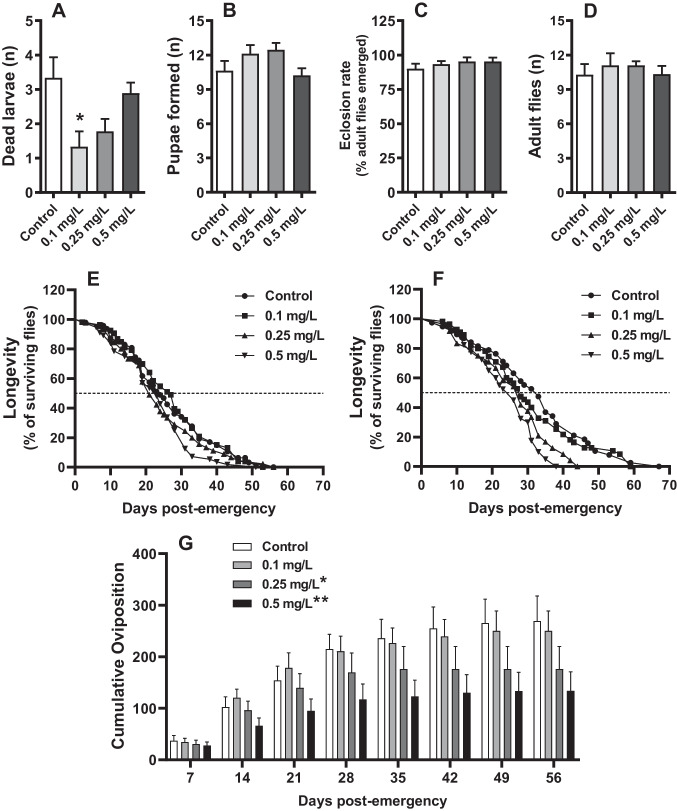
Effects of chronic exposure to Boral® 500 SC (SULF) on development, longevity, and female oviposition in *D. melanogaster*. (**A**) Larval mortality in flies exposed from the embryonic stage to 0.1, 0.25, or 0.5 mg/L of the active ingredient SULF or control conditions. (**B**) Pupation rate, expressed as the number of larvae reaching the pupal stage. (**C**) Adult emergence rate, expressed as the percentage of pupae that successfully emerged as adults. (**D**) Total number of emerged adults per vial**.** Statistical analyses for panels A–D were performed using one-way ANOVA followed by Tukey’s post hoc test. Adult longevity of (**E**) female and (**F**) male flies continuously exposed to Boral® 500 SC from the egg stage. Survival curves were analyzed using the log-rank (Mantel–Cox) test for trend. (**G**) Cumulative female oviposition over time. Oviposition was analyzed using repeated-measures ANOVA followed by Tukey’s post hoc test, and results are expressed as total eggs laid per female. Asterisks (*) indicate statistically significant differences (*P* < 0.05) compared with the control group

We first evaluated the effects of chronic exposure to the commercial formulation Boral® 500 SC on *D. melanogaster* development at relevant concentrations close to those found in natural environments following applications (Thorngren et al. 2017). At the lowest concentration tested (0.1 mg/L), exposure resulted in a significant reduction in larval mortality (Fig. 1A). However, no significant effects were observed on pupal formation (Fig. 1B), pupal-to-adult transition rate (Fig. 1C), or total adult emergence (Fig. 1D) at any of the tested concentrations. Likewise, developmental timing remained unaltered, as no differences were detected in the duration of the larval or pupal stages among experimental groups (data not shown). In contrast, longevity analysis revealed a significant reduction in mean lifespan in both female (*P* = 0.0276) and male (*P* = 0.0002) flies (Fig. 1E and Fig. 1F, respectively). However, no sex-related differences in mortality were observed when females and males exposed to the same herbicide concentration were compared using the log-rank (Mantel–Cox) test. Reproductive performance was also adversely affected. Females exposed to the two highest concentrations of Boral® 500 SC displayed a significant reduction in oviposition across the lifespan (Fig. 1G). Repeated-measures ANOVA indicated a significant effect of herbicide treatment (*P* = 0.0006), and Tukey’s post hoc analysis confirmed that females exposed to 0.25 mg/L and 0.5 mg/L laid significantly fewer eggs than controls (*P* = 0.0275 and *P* = 0.0051, respectively).

### Effects of Boral® 500 SC (SULF) on locomotor performance in *D. melanogaster*

**Fig. 2:**
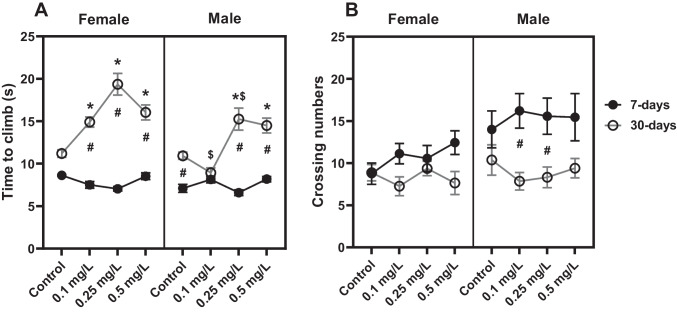
Age-dependent locomotor impairment induced by chronic exposure to Boral® 500 SC (SULF) in *D. melanogaster*. (**A**) Climbing performance assessed by the negative geotaxis assay, expressed as the time required to reach the upper portion of a vertical glass column. (**B**) Exploratory locomotor activity evaluated using the open-field assay, expressed as the number of grid crossings within a 30-s interval. Flies were continuously exposed to the herbicide from the embryonic stage, and after adult emergence were segregated by sex and maintained under their respective exposure conditions until behavioral assessment at 7 or 30 days post-emergence. Statistical analyses were performed using three-way ANOVA (factors: concentration, age, and sex; summarized in Table 1), followed by Tukey’s multiple-comparison post hoc test. Asterisks (*) indicate *P* < 0.05 for herbicide-exposed groups compared with their respective controls; hash symbols (#) indicate *P* < 0.05 for 30-day-old compared with 7-day-old flies; dollar signs ($) indicate *P* < 0.05 for male compared with female groups

Locomotor performance was first evaluated using the negative geotaxis assay, in which climbing ability was assessed by measuring the time required for adult flies to reach the upper portion of a vertical glass column. Chronic exposure to Boral® 500 SC resulted in a significant impairment of climbing performance, as evidenced by an increased climbing time in exposed flies (Fig. 2A). This effect was observed in females (*P* < 0.0002) and males (*P* < 0.0250) at all tested concentrations, with the exception of males exposed to 0.1 mg/L. Notably, these locomotor deficits were restricted to older individuals, as no significant effects were detected in 7-day-old flies. Furthermore, three-way ANOVA revealed a significant interaction among concentration, age, and sex, with the exception of the concentration × sex interaction, which was not statistically significant (Table 1).

**Table 1:** Statistical values from the three-way ANOVA. The variation factors were defined as herbicide concentration (control, 0.1, 0.25 and 0.5 mg/L), sex (females and males), and age (7 and 30 days) of the animals

	Negative Geotaxis	Open Field	Weight	Total Proteins	Total Carbohydrates	Total Lipids	Dehydration Resistance	Starvation Resistance	OS Resistance
Conc.	F (3, 25) = 17.3 *P* < 0.0001****	F (3, 48) = 0.14 *P* = 0.9353	F (3, 93) = 1.21 *P* = 0.3092	F (3, 12) = 1.54 *P* = 0.2069	F (3, 13) = 5.53 *P* = 0.0013**	F (3, 13) = 1.55 *P* = 0.2023	F (3, 76) = 0.80 *P* = 0.4941	F (3, 74) = 0.72 *P* = 0.5428	F (3, 75) = 4.02 *P* = 0.0104*
Sex	F (1, 25) = 28.3 *P* < 0.0001****	F (1, 48) = 8.99 *P* = 0.0028*	F (1, 93) = 5.60 *P* = 0.0200*	F (1, 12) = 0.31 *P* = 0.5735	F (1, 13) = 11.1 *P* = 0.0011**	F (1, 13) = 35.4 *P* < 0.0001****	F (1,76) = 1.60 *P* = 0.2096	F (1, 74) = 6.38 *P* = 0.0136*	F (1, 75) = 23.1 *P* < 0.0001****
Age	F (1, 25) = 374 *P* < 0.0001****	F (1, 48) = 24.5 *P* < 0.0001****	F (1, 93) = 2.51 *P* = 0.1163	F (1, 12) = 1.59 *P* = 0.2092	F (1, 13) = 2.70 *P* = 0.1026	F (1, 13) = 31.2 *P* < 0.0001****	F (1, 76) = 10.7 *P* = 0.0016**	F (1, 74) = 44.5 *P* = 0.0001****	F (1, 75) = 12.2 *P* = 0.0008***
Conc. × Sex	F (3, 25) = 2.02 *P* = 0.1112	F (3, 48) = 0.01 *P* = 0.9524	F (3, 93) = 1.42 *P* = 0.2413	F (3, 12) = 0.03 *P* = 0.9924	F (3, 13) = 5.55 *P* = 0.0013**	F (3, 13) = 1.40 *P* = 0.2433	F (3, 76) = 0.24 *P* = 0.8638	F (3, 74) = 0.20 *P* = 0.8929	F (3, 75) = 0.93 *P* = 0.4286
Conc. × Age	F (3, 25) = 27.4 *P* < 0.0001****	F (3, 48) = 1.18 *P* = 0.3146	F (3, 93) = 0.49 *P* = 0.6961	F (3, 12) = 2.13 *P* = 0.0997	F (3, 13) = 3.22 *P* = 0.0247*	F (3, 13) = 1.89 *P* = 0.1329	F (3, 76) = 0.90 *P* = 0.4421	F (3, 74) = 1.18 *P* = 0.3203	F (3, 75) = 0.38 *P* = 0.7673
Sex × Age	F (1, 25) = 15.9 *P* < 0.0001****	F (1, 48) = 4.86 *P* = 0.0279*	F (1, 93) = 1.04 *P* = 0.3105	F (1, 12) = 0.007 *P* = 0.9300	F (1, 13) = 1.48 *P* = 0.2252	F (1, 13) = 15.6 *P* = 0.0001***	F (1, 76) = 7.63 *P* = 0.0072**	F (1, 74) = 0.43 *P* = 0.5097	F (1, 75) = 0.01 *P* = 0.9155
C. × Sex × Age	F (3, 25) = 6.83 *P* = 0.0002***	F (3, 48) = 0.31 *P* = 0.8172	F (3, 93) = 2.34 *P* = 0.0783	F (3, 12) = 2.57 *P* = 0.0569	F (3, 13) = 9.22 *P* < 0.0001****	F (3, 13) = 4.84 *P* = 0.0031**	F (3, 76) = 0.78 *P* = 0.5045	F (3, 74) = 0.99 *P* = 0.4017	F (3, 75) = 0.65 *P* = 0.5811

Exploratory locomotor activity was further evaluated using the open-field assay, in which spontaneous locomotion was quantified as the number of grid crossings within a 30-s observation period. In this assay, 30-day-old males exhibited a significant reduction in locomotor activity compared with 7-day-old males when exposed to 0.1 mg/L (*P* = 0.0054) and 0.25 mg/L (*P* = 0.0337) of herbicide (Fig. 2B). In contrast, no significant alterations were detected in females at any tested concentration. Three-way ANOVA identified sex and age as significant main effects, as well as a significant Sex × Age interaction (Table 1).

### Effects of Boral® 500 SC (SULF) on metabolic parameters in *D. melanogaster*

**Fig. 3:**
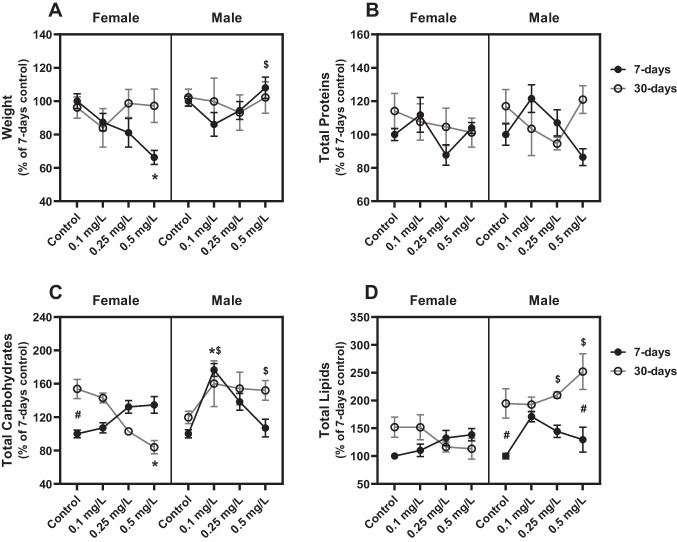
Age- and sex-dependent effects of chronic exposure to Boral® 500 SC (SULF) on metabolic parameters in *D. melanogaster*. Flies were exposed to the herbicide from the egg stage, and after adult emergence were separated by sex into different vials and continuously maintained under exposure until day 7 or day 30, when metabolic analyses were performed. (**A**) Total body weight, (**B**) total protein content, (**C**) total carbohydrate levels, and (**D**) total lipid levels. Data presented in relation to the 7-day percentage control. Statistical analysis was performed using three-way ANOVA (summarized in Table 1), followed by Tukey’s multiple comparisons post hoc test. * indicates *P* < 0.05 for herbicide-exposed groups compared with their respective controls; # indicates *P* < 0.05 for 30-day-old groups compared with 7-day-old groups; $ indicates *P* < 0.05 for males compared with females

Metabolic homeostasis was evaluated by assessing body weight and total protein, carbohydrate, and lipid contents in flies chronically exposed to Boral® 500 SC. A significant reduction in body weight was observed in 7-day-old females exposed to 0.5 mg/L compared with age-matched controls (*P* = 0.0167). In contrast, 7-day-old males exposed to the same concentration exhibited higher body weight than females within the same treatment group (*P* = 0.0232) (Fig. 3A). Consistent with these findings, sex emerged as a significant factor in the three-way ANOVA (Table 1).

Total protein levels were not significantly affected by herbicide exposure, as no main effects or interactions were detected by three-way ANOVA or subsequent post hoc analyses (Fig. 3B).

In contrast, carbohydrate levels was selectively altered. In control groups, 30-day-old females exhibited lower carbohydrate levels than 7-day-old females (*P* = 0.0380), an age-related difference that was not observed in herbicide-exposed females (Fig. 3C). Notably, 30-day-old females exposed to 0.5 mg/L showed a significant reduction in total carbohydrate levels compared with their age-matched controls (*P* = 0.0035).

Importantly, sex-dependent effects were also evident in carbohydrate content. In fact, seven-day-old males exposed to 0.1 mg/L displayed increased carbohydrate levels compared with females from the same treatment group (*P* = 0.0016), as well as relative to their respective controls (*P* < 0.0001). Similarly, 30-day-old males exposed to 0.5 mg/L exhibited higher carbohydrate levels than females of the same age and treatment (*P* = 0.0111) (Fig. 3C). Three-way ANOVA indicated that concentration was the primary factor driving carbohydrate alterations, whereas age and the Sex × Age interaction were not significant (Table 1).

Alterations in lipid content were restricted to males. Age-related differences were observed between control males (*P* = 0.0030) and those exposed to 0.5 mg/L (*P* < 0.0001). In addition, 30-day-old males exposed to 0.25 mg/L (*P* = 0.0188) and 0.5 mg/L (*P* < 0.0001) exhibited significantly higher lipid levels than females from the corresponding groups (Fig. 3D). Three-way ANOVA identified age and sex as significant main effects, along with Sex × Age and Sex × Age × Concentration interactions (Table 1).

### Effects of Boral® 500 SC (SULF) on stress resistance in *D. melanogaster*

**Fig. 4:**
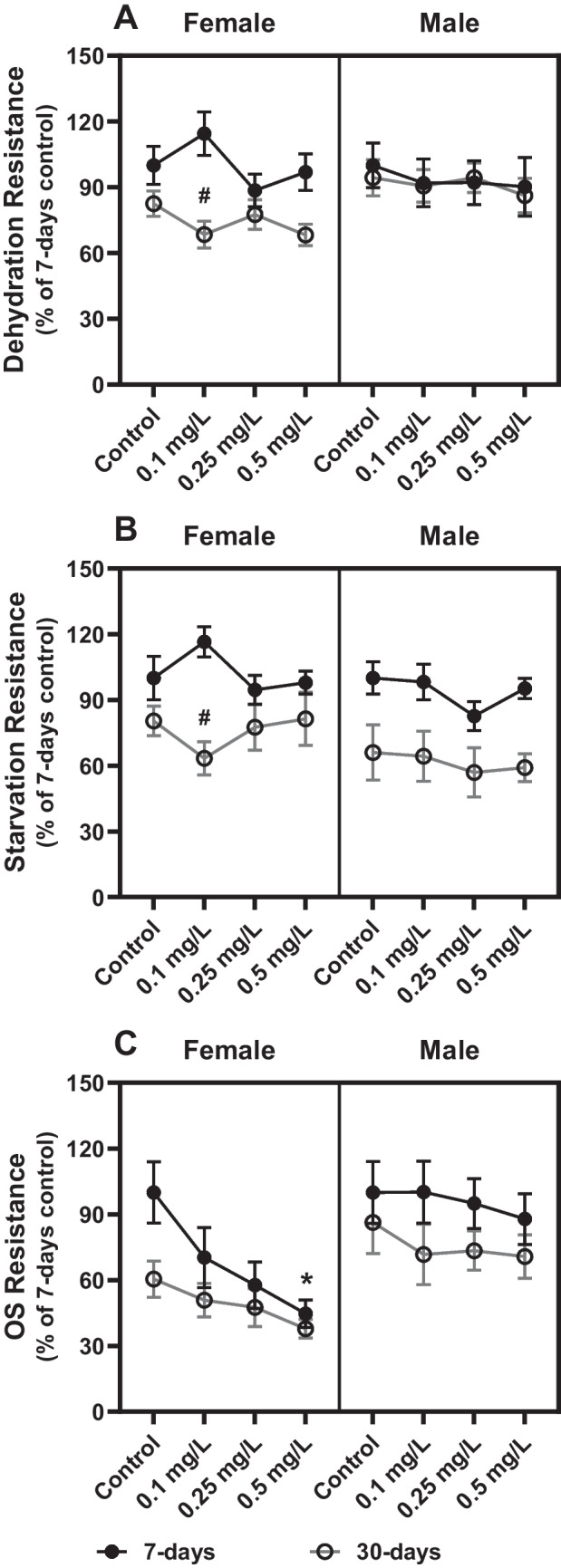
Stress resistance in *D. melanogaster* chronically exposed to Boral® 500 SC (SULF). Flies were exposed to the herbicide from the egg stage and, after adult emergence, were separated by sex and maintained under their respective treatments until 7 or 30 days of age, when stress resistance assays were conducted. (**A**) Dehydration resistance, assessed under complete water deprivation; (**B**) starvation resistance, evaluated under nutrient deprivation with access to agar as a moisture source; and (**C**) oxidative stress resistance, assessed following exposure to paraquat. Survival data were plotted as XY curves, and the area under the curve was extracted for three-way ANOVA analysis (summarized in Table 1), followed by Tukey’s multiple comparisons post hoc test. Data represented as a percentage of the 7-day average control. * indicates *P* < 0.05 for herbicide-exposed groups compared with their respective controls; # indicates *P* < 0.05 for 30-day-old groups compared with 7-day-old groups

To determine whether chronic exposure to Boral® 500 SC alters stress resistance in *D. melanogaster*, flies were subjected to dehydration, starvation, and oxidative stress challenges. Under dehydration and starvation conditions, significant age-dependent effects were observed exclusively in females exposed to 0.1 mg/L. Specifically, differences between 7- and 30-day-old females were detected in the dehydration resistance assay (*P* = 0.0299; Fig. 4A) and in the starvation resistance assay (*P* = 0.0064; Fig. 4B). No additional significant differences were identified by post hoc analyses in these assays. Three-way ANOVA revealed that, in the dehydration resistance test, age emerged as the only significant variable, along with a significant Sex × Age interaction. In contrast, in the starvation resistance assay, sex and age were significant main factors (Table 1). In turn, resistance to oxidative stress was assessed by exposing flies to paraquat (20 mM). In this assay, females chronically exposed to the highest herbicide concentration (0.5 mg/L) exhibited reduced survival compared with control females (*P* = 0.0198; Fig. 4C). Three-way ANOVA identified herbicide concentration, sex, and age as significant factors (Table 1).

### Principal component analysis of endpoints measured in *D. melanogaster* exposed to Boral® 500 SC (SULF)

To explore potential relationships among the reproductive, behavioral, metabolic, and stress-related endpoints evaluated in *D. melanogaster* chronically exposed to Boral® 500 SC, PCA was performed separately according to sex and age.

**Fig. 5:**
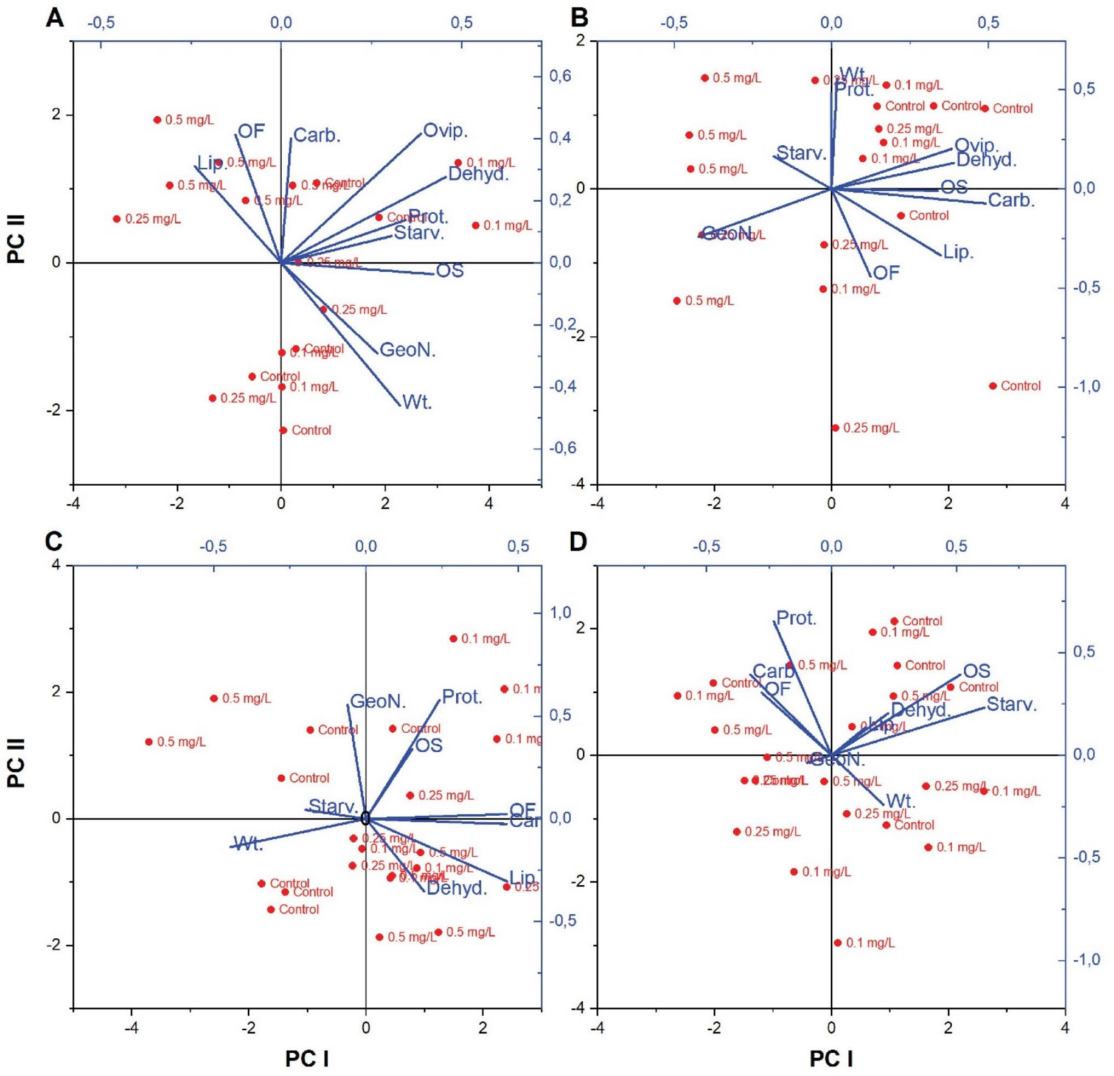
Principal component analysis (PCA) plots derived from female reproduction, locomotor performance, metabolic parameters, and stress resistance in *D. melanogaster* chronically exposed to Boral® 500 SC (SULF). The graphs depict mean scores and the orientation of the primary variables contributing to the observed alterations. Panels represent (**A**) 7 day old females, (**B**) 30 day old females, (**C**) 7 day old males, and (**D**) 30 day old males. Data were normalized to their respective controls. Biplots of PC1 and PC2 were generated to visualize correlations among variables across sex and age groups. Variables included reproductive output (Ovip.), locomotor behavior assessed by negative geotaxis (GeoN.) and open field activity (OF), metabolic endpoints comprising body weight (Wt.), total protein (Prot.), carbohydrate (Carb.), and lipid (Lip.) levels, as well as stress resistance parameters: dehydration (Dehyd.), starvation (Starv.), and oxidative stress (OS).In males, PCA revealed a comparable pattern. In 7-day-old individuals (Fig.  5 C), body weight was negatively correlated with lipid and carbohydrate levels and with exploratory locomotor activity assessed by the open-field test. In 30-day-old males (Fig. 5D), this inverse association was maintained and accompanied by a positive correlation among stress resistance parameters, including starvation, dehydration, and oxidative stress resistance

In 7-day-old females (Fig. 5A), body weight and locomotor performance assessed by the negative geotaxis assay were oriented in the opposite direction to total lipid and carbohydrate contents, as well as exploratory activity in the open-field test, indicating a negative correlation between locomotor performance and energetic reserves. In 30-day-old females (Fig. 5B), negative geotaxis was negatively correlated with oviposition, resistance to dehydration and oxidative stress, and total carbohydrate levels, reinforcing the association between locomotor capacity and metabolic and physiological resilience at later life stages. Notably, in both female age groups, locomotor performance consistently aligned with variations in energy reserves.

Overall, the multivariate analyses demonstrate coherent and sex-specific correlations among behavioral performance, energy metabolism, reproductive output, and stress resilience, highlighting the integrative nature of the physiological disturbances induced by chronic exposure to Boral® 500 SC.

## Discussion

In this study, we demonstrate that chronic exposure to sublethal concentrations of the commercial herbicide formulation Boral® 500 SC (SULF) significantly reduces the longevity of *D. melanogaster* when exposure occurs from the embryonic stage onward**.** In contrast, at the concentrations tested, no detrimental effects were detected on early developmental endpoints, including larval survival, pupation rate, or adult emergence. Despite that**,** marked sublethal effects became evident during adulthood, encompassing impairments in climbing performance, as well as alterations in body weight and in the levels of total proteins, carbohydrates, and lipids. Importantly, the majority of these effects exhibited a clear dependence on herbicide concentration, sex, and adult age, highlighting a complex pattern of susceptibility. In addition to behavioral and metabolic disturbances, exposure to Boral® 500 SC compromised the ability of flies to withstand oxidative stress, indicating reduced stress resilience. Finally, multivariate analysis using PCA revealed robust correlations between metabolic status and behavioral performance, underscoring the central role of energy reserve disruption in mediating the behavioral phenotypes observed in Boral® 500 SC-exposed flies.

### Development, longevity, and locomotion

To evaluate the effects of Boral® 500 SC on multiple biological endpoints in *D. melanogaster*, we first focused on developmental parameters, which represent sensitive indicators of early-life toxicity. At concentrations close to those found in natural environments following application, no increases in mortality or alterations in developmental timing were detected (Fig. 1A–1D). These findings indicate that early developmental macro-parameters were less responsive to exposure at the concentrations tested, suggesting that the sublethal effects resulting from chronic and cumulative herbicide ingestion may not become evident during the relatively short larval period but may instead emerge later in adult life.

In contrast, clear adverse effects emerged at the adult stage, as demonstrated by the significant reduction in longevity observed in flies of both sexes exposed to Boral® 500 SC (Fig. 1E and Fig. 1F). Decreased lifespan has been consistently reported in insects exposed to pesticides and other chronic stressors (Tadei et al. 2019; Belyi et al. 2020) and is commonly associated with a progressive decline in the organism’s capacity to cope with physiological challenges. This decline is thought to arise from reduced expression and efficiency of cellular repair mechanisms and antioxidant defenses (Yusuf et al. 2024), processes that are intrinsically linked to aging and have been extensively characterized in *D. melanogaster* (Jones and Grotewiel 2011). Under chronic toxic exposure, these age-related vulnerabilities are likely exacerbated, accelerating functional deterioration. Mitochondrial dysfunction may represent a key mechanistic link underlying the observed reduction in lifespan, as oxidative imbalance and impaired cellular repair can compromise mitochondrial bioenergetics. Such impairments are particularly detrimental under conditions of sustained cellular stress, where energy demands are elevated. In line with this interpretation, mitochondrial dysfunction has been strongly associated with aging-related pathologies, reduced stress resistance, and shortened lifespan (Tower 2015; Srivastava 2017).

Aging in *D. melanogaster* is also accompanied by a progressive decline in locomotor performance, which has been attributed to functional impairments in the nervous and/or muscular systems (Jones and Grotewiel 2011). Consistent with this well-established phenotype, our results revealed an age-related reduction in climbing ability (Fig. 2A). Notably, chronic exposure to Boral® 500 SC exacerbated this decline, significantly impairing negative geotaxis performance in both males and females. Three-way ANOVA confirmed significant contributions of herbicide concentration, sex, and age, underscoring the multifactorial nature of the locomotor deficits observed. In contrast, exploratory locomotor behavior assessed by the open-field assay was not directly affected by herbicide exposure, reflecting primarily an age-dependent decline in males (Fig. 2B) and intrinsic sex-related differences when compared with females (Table 1). Sex-specific responses to toxic stimuli are well documented in *D. melanogaster* and are largely attributed to genetic, hormonal, and metabolic differences underlying sexual dimorphism (Tricoire et al. 2009; Niveditha et al. 2017; Videlier et al. 2021; Gomes et al. 2023; Yusuf et al. 2024).

### Metabolic parameters

Alterations in development, longevity, and behavior in *D. melanogaster* are frequently associated with underlying changes in metabolic homeostasis. In the present study, chronic exposure to Boral® 500 SC induced measurable modifications in body weight and in total carbohydrate, and lipid contents, with these effects being strongly modulated by herbicide concentration, age, and sex (Table 1). Such multifactorial dependence underscores the complexity of the metabolic responses elicited by low-dose, chronic herbicide exposure. The reduction in body weight observed in 7-day-old females exposed to 0.5 mg/L, relative to age-matched controls, does not appear to reflect a uniform toxic effect across sexes, as males from the same treatment group exhibited significantly higher body weight when compared with females (Fig. 3A). This sex-dependent divergence suggests differential metabolic allocation strategies rather than a generalized impairment of growth or feeding behavior. Supporting this interpretation, previous studies have reported increases in body weight and lipid content (Wilkens et al. 2019) as well as elevated total protein levels (da Silva et al. 2021) in tadpoles exposed to Boral® 500 SC. Although sexual dimorphism was not addressed in those studies, our findings indicate that sex-specific metabolic regulation plays a critical role in shaping organismal responses to Boral® 500 SC exposure. In *D. melanogaster*, energy management differs substantially between males and females, particularly under nutritional or environmental stress. For instance, males tend to exhibit shorter longevity despite preserving lipid reserves, whereas females often display extended lifespan accompanied by reduced lipid stores (Bak et al. 2023), a pattern remarkably consistent with our observations for total lipid content in herbicide-exposed flies (Fig. 3D). These findings support the notion that metabolic parameters both influence and reflect sex-specific life-history strategies, especially those linked to reproduction. From a reproductive standpoint, females allocate substantial metabolic resources to oogenesis, supplying eggs with not only genetic material but also energy-dense substrates essential for embryonic development, whereas males primarily invest in gamete production with comparatively lower energetic costs (reviewed by Shingleton and Vea 2023). As highlighted in that review, sex-specific metabolic regulation involves distinct endocrine and molecular pathways, including lipolysis in the male fat body mediated by the triglyceride lipase *bummer* and, in females, lipid digestion, absorption, and synthesis in the midgut regulated by ecdysone and juvenile hormone signaling. Although these pathways were not directly assessed in the present study, our data suggest that chronic exposure to Boral® 500 SC may interfere with energy allocation processes that are inherently sex dependent. Nevertheless, it is not currently possible to directly link the observed metabolic alterations to endocrine disruption mechanisms in flies exposed to Boral® 500 SC. This represents a relevant regulatory and mechanistic gap, particularly given that potential endocrine-disrupting effects of SULF remain insufficiently explored in animal models, despite regulatory assessments by the U.S. EPA indicating low acute risk to *Apis mellifera* (Sinclair et al. 2014).

In this context, the herbicide’s mode of action may influence energy metabolism even at sublethal concentrations. In a previous study, we demonstrated that acute exposure to Boral® 500 SC increases energetic demand in *D. melanogaster* (Gayer et al. 2025). Such increased demand is likely driven by detoxification processes, activation of antioxidant defenses, and repair of oxidative damage, all of which require substantial energy investment (Gusarov et al. 2017; Riahi et al. 2019). Furthermore, inhibition of the protoporphyrinogen oxidase (PPOX) enzyme diverts tricarboxylic acid cycle intermediates toward heme biosynthesis, potentially compromising cellular ATP production due to the diversion of these intermediates for heme formation rather than energy generation (Homedan et al. 2014; Burch et al. 2018). The combined effects of increased energetic demand and reduced metabolic efficiency are likely reflected in the impaired reproductive output observed in exposed females (Fig. 1G). As previously discussed, female reproduction is an energetically expensive process, and under conditions of sustained metabolic stress, investment in egg production may be selectively reduced to preserve somatic maintenance. In support of this interpretation, PCA revealed a strong correlation between oviposition and carbohydrate levels in 30-day-old females (Fig. 5B), indicating that carbohydrate availability may be a key determinant of reproductive performance under chronic herbicide exposure. Finally, locomotor performance may also be indirectly affected by alterations in energy reserves, as insufficient or misallocated metabolic substrates can compromise neuromuscular function. Consistently, PCA demonstrated robust correlations between behavioral endpoints and metabolic parameters across sexes and age groups (Fig. 5), reinforcing the concept that behavioral impairments observed in herbicide-exposed flies are tightly linked to disruptions in energetic homeostasis.

### Stress resistance

Several studies have highlighted the potential negative effects of pesticide exposure on populations of diverse insect species (Janousek et al. 2023; Quandahor et al. 2024). Importantly, beyond direct effects on mortality, pesticide exposure may critically impair the ability of insects to withstand additional environmental stressors, thereby compromising long-term survival. In this context, our results demonstrate that female *D. melanogaster* exposed to 0.5 mg/L of the commercial formulation Boral® 500 SC exhibit increased susceptibility to paraquat-induced oxidative stress, particularly at 7 days of age (Fig. 4C). This finding indicates that early-adult females subjected to chronic herbicide exposure are more vulnerable to secondary oxidative challenges, suggesting a reduced physiological buffering capacity. Such heightened sensitivity implies that these females may experience exacerbated damage under scenarios of cumulative or combined exposure to multiple oxidative stressors, a condition that is ecologically realistic in agricultural landscapes. This vulnerability has direct consequences for individual survival and shortens the effective reproductive window, thereby limiting egg production and, ultimately, the capacity for population maintenance. Notably, this scenario is further aggravated by the herbicide-induced reduction in female oviposition observed in the present study (Fig. 1G), reinforcing the potential for long-term demographic impacts.

Taken together, these findings underscore the necessity of incorporating multi-stress paradigms into ecotoxicological assessments, as evaluating pesticide exposure in isolation may substantially underestimate ecological risk. The interaction between chemical exposure and additional stress factors can overwhelm organismal resilience, providing a plausible mechanistic explanation for the widespread declines observed in insect populations, including the alarming reductions reported for pollinator species (Rhodes 2018; Janousek et al. 2023; Ellis et al. 2025). Given the essential ecological services provided by pollinators—ranging from the maintenance of plant and animal biodiversity to their substantial economic value in agricultural systems—such combined stress effects represent a critical concern for environmental sustainability and food security.

## Conclusion

Our study investigated the toxicological consequences of chronic exposure of *D. melanogaster* to the commercial formulation Boral® 500 SC (SULF) from the earliest developmental stages, at concentrations close to those found in natural environments following application. While early developmental endpoints—including larval survival, pupation, and adult emergence—were largely unaffected, chronic exposure induced a consistent pattern of biologically relevant sublethal effects in adult flies. These included reduced longevity, decreased female oviposition, impairment of climbing locomotor performance, sex- and age-dependent metabolic dysregulation, and decreased resistance to oxidative stress, particularly in females. Notably, the commercial formulation has been reported to exert toxicological effects on *D. melanogaster*, while the active ingredient SULF is classified as “practically non-toxic” for *Apis mellifera* based on acute mortality assays. These findings underscore the importance of considering formulation components and chronic sublethal effects in ecological risk assessment. Incorporating functionally relevant endpoints—including behavioral, metabolic, and stress-resilience parameters—is essential for accurately predicting the impacts of herbicides on non-target insect species and for informing more comprehensive regulatory evaluations. Collectively, these results support the hypothesis that chronic exposure to sublethal concentrations of Boral® 500 SC induces biologically relevant effects across multiple physiological and behavioral endpoints in *D. melanogaster*.

## Data Availability

Data will be made available on request.

## References

[CR1] Aufderheide J, Kranzfelder J (1996) Sulfentrazone technical: honey bee acute contact LD50: Lab project number: A95–4228: J9507010: F616. Unpublished study prepared by Toxikon Environmental Sciences, p 31

[CR2] Bak NK, Rohde PD, Kristensen TN (2023) Strong sex-dependent effects of malnutrition on life-and healthspan in *Drosophila melanogaster*. Insects 15(1):9. 10.3390/insects15010009 38249015 10.3390/insects15010009 PMC10816799

[CR3] Belyi AA, Alekseev AA, Fedintsev AY, Balybin SN, Proshkina EN, Shaposhnikov MV, Moskalev AA (2020) The resistance of *Drosophila melanogaster* to oxidative, genotoxic, proteotoxic, osmotic stress, infection, and starvation depends on age according to the stress factor. Antioxidants 9(12):1239. 10.3390/antiox9121239 33297320 10.3390/antiox9121239 PMC7762242

[CR4] Bradford MM (1976) A rapid and sensitive method for the quantitation of microgram quantities of protein utilizing the principle of protein-dye binding. Anal Biochem 72(1–2):248–254. 10.1016/0003-2697(76)90527-3 942051 10.1016/0003-2697(76)90527-3

[CR5] Burch JS, Marcero JR, Maschek JA, Cox JE, Jackson LK, Medlock AE, Phillips JD, Dailey HA Jr (2018) Glutamine via α-ketoglutarate dehydrogenase provides succinyl-CoA for heme synthesis during erythropoiesis. Blood 132(10):987–998. 10.1182/blood-2018-01-829036 29991557 10.1182/blood-2018-01-829036 PMC6128084

[CR6] da Silva PR, Borges-Martins M, Oliveira GT (2021) *Melanophryniscus admirabilis* tadpoles’ responses to sulfentrazone and glyphosate-based herbicides: An approach on metabolism and antioxidant defenses. Environ Sci Pollut Res 28(4):4156–4172. 10.1007/s11356-020-10654-x 10.1007/s11356-020-10654-x 32935212

[CR7] Ellis EE, Campbell SA, Edmondson JL (2025) Drivers of nocturnal and diurnal pollinating insect declines in urban landscapes. Proc Biol Sci 292:2052. 10.1098/rspb.2025.0102 10.1098/rspb.2025.0102 PMC12324870 40763813

[CR8] Feany MB, Bender WW (2000) A Drosophila model of Parkinson’s disease. Nature 404(6776):394–398. 10.1038/35006074 10746727 10.1038/35006074

[CR9] Freitas JS, Teresa FB, de Almeida EA (2017) Influence of temperature on the antioxidant responses and lipid peroxidation of two species of tadpoles (*Rhinella schneideri* and *Physalaemus nattereri*) exposed to the herbicide sulfentrazone (Boral 500SC®). Comp Biochem Physiol C Toxicol Pharmacol 197:32–44. 10.1016/j.cbpc.2017.04.005 28457947 10.1016/j.cbpc.2017.04.005

[CR10] Gayer MC, Bianchini MC, Carriço MRS, Paz MEG, Nogueira CL, Denardin ELG, Puntel RL, Roehrs R (2025) Boral® 500 SC (sulfentrazone) induces accumulation of heme synthesis intermediates and changes in locomotor behavior and metabolic markers in *Drosophila melanogaster*. Chemosphere 380:144468. 10.1016/j.chemosphere.2025.144468 40344814 10.1016/j.chemosphere.2025.144468

[CR11] Gomes KK, dos Santos AB, dos Anjos JS, Leandro LP, Mariano MT, Pinheiro FL, Farina M, Franco JL, Posser T (2023) Increased iron levels and oxidative stress mediate age-related impairments in male and female drosophila melanogaster. Oxid Med Cell Longev 2023(1):7222462. 10.1155/2023/7222462 37333463 10.1155/2023/7222462 PMC10275690

[CR12] Gusarov I, Pani B, Gautier L, Smolentseva O, Eremina S, Shamovsky I, Katkova-Zhukotskaya O, Mironov A, Nudler E (2017) Glycogen controls *Caenorhabditis elegans* lifespan and resistance to oxidative stress. Nat Commun 8(1):1–12. 10.1038/ncomms15868 28627510 10.1038/ncomms15868 PMC5481799

[CR13] Homedan C, Laafi J, Schmitt C, Gueguen N, Lefebvre T, Karim Z, Desquiret-Dumas V, Wetterwald C, Deybach JC, Gouya L, Puy H, Reynier P, Malthièry Y (2014) Acute intermittent porphyria causes hepatic mitochondrial energetic failure in a mouse model. Int J Biochem Cell Biol 51:93–101. 10.1016/j.biocel.2014.03.032 24727425 10.1016/j.biocel.2014.03.032

[CR14] Janousek WM, Douglas MR, Cannings S, Clément MA, Delphia CM, Everett JG, Hatfield RG, Keinath DA, Koch JBU, McCabe LM, Mola JM, Ogilvie JE, Rangwala I, Richardson LL, Rohde AT, Strange JP, Tronstad LM, Graves TA (2023) Recent and future declines of a historically widespread pollinator linked to climate, land cover, and pesticides. Proc Natl Acad Sci U S A 120(5):e2211223120. 10.1073/pnas.2211223120 36689649 10.1073/pnas.2211223120 PMC9945941

[CR15] Jiang J, Wang L, Zhang C, Zhao X (2022) Health risks of sulfentrazone exposure during zebrafish embryo-larvae development at environmental concentration. Chemosphere 288:132632. 10.1016/j.chemosphere.2021.132632 34687687 10.1016/j.chemosphere.2021.132632

[CR16] Jones MA, Grotewiel M (2011) Drosophila as a model for age-related impairment in locomotor and other behaviors. Exp Gerontol 46(5):320–325. 10.1016/j.exger.2010.08.012 20800672 10.1016/j.exger.2010.08.012 PMC3021004

[CR17] Li M, Ma X, Saleem M, Wang X, Sun L, Yang Y, Zhang Q (2020) Biochemical response, histopathological change and DNA damage in earthworm (*Eisenia fetida*) exposed to sulfentrazone herbicide. Ecol Indic 115:106465. 10.1016/j.ecolind.2020.106465

[CR18] Lin YC, Zhang M, Chang YJ, Kuo TH (2023) Comparisons of lifespan and stress resistance between sexes in *Drosophila melanogaster*. Heliyon 9(8):e18178. 10.1016/j.heliyon.2023.e18178 37576293 10.1016/j.heliyon.2023.e18178 PMC10415617

[CR19] Martinez CO, Silva CMM, Fay EF, Maia ADHN, Abakerli RB, Durrant LR (2008) Degradation of the herbicide sulfentrazone in a Brazilian typic hapludox soil. Soil Biol Biochem 40(4):879–886. 10.1016/j.soilbio.2007.10.016

[CR20] Mesak C, de Campos RP, de Melo MA, Mendes BO, Malafaia G (2018) Behavioral response and dynamics of *Eisenia fetida* hemocytes exposed to environmentally relevant concentration of sulfentrazone. Enviro Sci Pollut Res 25(30):30728–30736. 10.1007/s11356-018-3175-8 10.1007/s11356-018-3175-8 30220066

[CR21] Mesnage R, Antoniou MN (2018) Ignoring adjuvant toxicity falsifies the safety profile of commercial pesticides. Front Publ Health 5:361. 10.3389/fpubh.2017.00361 10.3389/fpubh.2017.00361 PMC5786549 29404314

[CR22] Möhring N, Ingold K, Kudsk P, Martin-Laurent F, Niggli U, Siegrist M, Studer B, Walter A, Finger R (2020) Pathways for advancing pesticide policies. Nat Food 1(9):535–540. 10.1038/s43016-020-00141-4 37128006 10.1038/s43016-020-00141-4

[CR23] Niveditha S, Deepashree S, Ramesh SR, Shivanandappa T (2017) Sex differences in oxidative stress resistance in relation to longevity in *Drosophila melanogaster*. J Comp Physiol B 187:899–909. 10.1007/s00360-017-1061-1 28261744 10.1007/s00360-017-1061-1

[CR24] Quandahor P, Kim L, Kim M, Lee K, Kusi F, Jeong IH (2024) Effects of agricultural pesticides on decline in insect species and individual numbers. Environments 11(8):182. 10.3390/environments11080182

[CR25] Raven PH, Wagner DL (2021) Agricultural intensification and climate change are rapidly decreasing insect biodiversity. PNAS 118(2):e2002548117. 10.1073/pnas.2002548117 33431564 10.1073/pnas.2002548117 PMC7812793

[CR26] Rhodes CJ (2018) Pollinator decline–an ecological calamity in the making? Sci Prog 101(2):121–160. 10.3184/003685018X15202512854527 29669627 10.3184/003685018X15202512854527 PMC10365189

[CR27] Riahi H, Brekelmans C, Foriel S, Merkling SH, Lyons TA, Itskov PM, Kleefstra T, Ribeiro C, van Rif RP, Kramer JM, Schenck A (2019) The histone methyltransferase G9a regulates tolerance to oxidative stress–induced energy consumption. PLoS Biol 17(3):e2006146. 10.1371/journal.pbio.2006146 30860988 10.1371/journal.pbio.2006146 PMC6413895

[CR28] Sánchez-Bayo F, Wyckhuys KA (2019) Worldwide decline of the entomofauna: a review of its drivers. Biol Conserv 232:8–27. 10.1016/j.biocon.2019.01.020

[CR29] Schneider D (2000) Using drosophila as a model insect. Nat Rev Genet 1(3):218–226. 10.1038/35042080 11252751 10.1038/35042080

[CR30] Shingleton AW, Vea IM (2023) Sex-specific regulation of development, growth and metabolism. Semin Cell Dev Biol 138:117–127. 10.1016/j.semcdb.2022.04.017 35469676 10.1016/j.semcdb.2022.04.017

[CR31] Sinclair G, Barrett M, Sappington K, Shamim M (2014) Preliminary ecological risk assessment for the registration review of sulfentrazone and proposed new uses on apples. https://www.regulations.gov/document/EPA-HQ-OPP-2009-0624-0017

[CR32] Srivastava S (2017) The mitochondrial basis of aging and age-related disorders. Genes 8(12):398. 10.3390/genes8120398 29257072 10.3390/genes8120398 PMC5748716

[CR33] Straub L, Strobl V, Neumann P (2020) The need for an evolutionary approach to ecotoxicology. Nat Ecol Evol 4(7):895–895. 10.1038/s41559-020-1194-6 32327743 10.1038/s41559-020-1194-6

[CR34] Straw EA, Brown MJ (2021) Co-formulant in a commercial fungicide product causes lethal and sub-lethal effects in bumble bees. Sci Rep 11(1):21653. 10.1038/s41598-021-00919-x 34741036 10.1038/s41598-021-00919-x PMC8571393

[CR35] Sudati JH, Vieira FA, Pavin SS, Dias GRM, Seeger RL, Golombieski R, Athayde ML, Soares FA, Rocha JBT, Barbosa NV (2013) *Valeriana officinalis* attenuates the rotenone-induced toxicity in *Drosophila melanogaster*. Neurotoxicology 37:118–126. 10.1016/j.neuro.2013.04.006 23639798 10.1016/j.neuro.2013.04.006

[CR36] Tadei R, Domingues CE, Malaquias JB, Camilo EV, Malaspina O, Silva-Zacarin EC (2019) Late effect of larval co-exposure to the insecticide clothianidin and fungicide pyraclostrobin in Africanized *Apis mellifera*. Sci Rep 9(1):3277. 10.1038/s41598-019-39383-z 30824742 10.1038/s41598-019-39383-z PMC6397237

[CR37] Thorngren JL, Harwood AD, Murphy TM, Hartz KEH, Fung CY, Lydy MJ (2017) Fate and risk of atrazine and sulfentrazone to nontarget species at an agriculture site. Environ Toxicol Chem 36(5):1301–1310. 10.1002/etc.3664 27779324 10.1002/etc.3664

[CR38] Tower J (2015) Mitochondrial maintenance failure in aging and role of sexual dimorphism. Arch Biochem Biophys 576:17–31. 10.1016/j.abb.2014.10.008 25447815 10.1016/j.abb.2014.10.008 PMC4409928

[CR39] Tricoire H, Battisti V, Trannoy S, Lasbleiz C, Pret AM, Monnier V (2009) The steroid hormone receptor EcR finely modulates Drosophila lifespan during adulthood in a sex-specific manner. Mech Ageing Dev 130(8):547–552. 10.1016/j.mad.2009.05.004 19486910 10.1016/j.mad.2009.05.004

[CR40] van Handel E (1985a) Rapid determination of glycogen and sugars in mosquitos. JAMCA 1(2):299–301 2906671

[CR41] van Handel E (1985b) Rapid determination of total lipids in mosquitos. JAMCA 1(2):302–304 2906672

[CR42] Videlier M, Rundle HD, Careau V (2021) Sex-specific genetic (co) variances of standard metabolic rate, body mass and locomotor activity in *Drosophila melanogaster*. J Evol Biol 34(8):1279–1289. 10.1111/jeb.13887 34107129 10.1111/jeb.13887

[CR43] Wang F, Chen S, Lv L, Wu S, Zhao Y, Liu X, Geng N, Tang T (2024) Metabolic perturbations in zebrafish (*Danio rerio*) larvae exposed to sulfentrazone and imidacloprid. Sci Total Environ 933:173150. 10.1016/j.scitotenv.2024.173150 38735312 10.1016/j.scitotenv.2024.173150

[CR44] Wāng Y (2025) *Drosophila melanogaster* as sentinel organism for hazard identification of environmental contaminants. J Hazard Mater 139600. 10.1016/j.jhazmat.2025.139600 10.1016/j.jhazmat.2025.139600 40845575

[CR45] Wilkens AL, Valgas AA, Oliveira GT (2019) Effects of ecologically relevant concentrations of Boral® 500 SC, Glifosato® Biocarb, and a blend of both herbicides on markers of metabolism, stress, and nutritional condition factors in bullfrog tadpoles. Environ Sci Pollut Res 26(23):23242–23256. 10.1007/s11356-019-05533-z 10.1007/s11356-019-05533-z 31190300

[CR46] Yusuf AO, Danborno B, Bauchi ZM, Sani D, Ndams IS (2024) Aging impaired locomotor and biochemical activities in *Drosophila melanogaster* Oregon R (fruit fly) model. Exp Gerontol 197:112593. 10.1016/j.exger.2024.112593 39326807 10.1016/j.exger.2024.112593

[CR47] Zagar C, Liebl R, Theodoridis G, Witschel M (2019) Protoporphyrinogen IX oxidase inhibitors. In: Jeschke P, Witschel M, Krämer W, Schirmer U (eds) Modern crop protection compounds, 3rd edn. Wiley-VCH, Weinheim, Germany, pp 173–211. 10.1002/9783527699261.ch3

